# Supplementary material for: The interactome of intact mitochondria by cross-linking mass spectrometry provides evidence for coexisting respiratory supercomplexes
Source: Mol Cell Proteomics. 2017 Dec 8;17(2):216–32. doi: 10.1074/mcp.RA117.000470 (PMC5795388; doi:10.1074/mcp.RA117.000470)
Supplement: Supplemental Data [file supp_RA117.000470_133922_0_supp_23978_fzffwf.zip › spectra_annotation/mito_spectra_annotation/467-1-2-1-11-1.pdf]

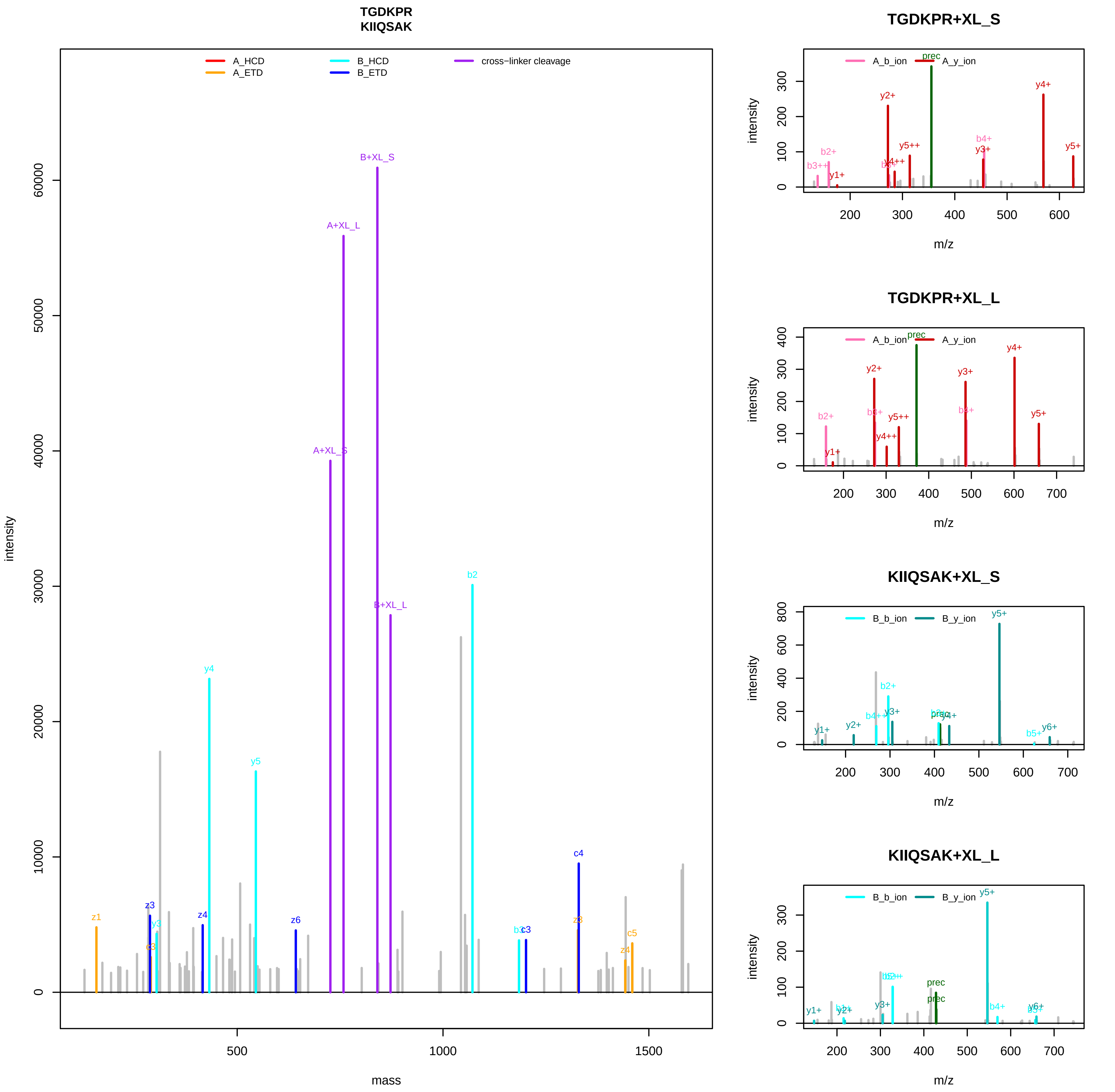

TGDKPR
KIIQSAK
A_HCD
A_ETD
B_HCD
B_ETD
cross−linker cleavage
B+XL_S
A+XL_L
A+XL_S
B+XL_L
b2
y4
y5
z1
z3
y3
c3
z4
z6
b3
c3
c4
z3
z4
c5
intensity
mass
TGDKPR+XL_S
A_b_ion
A_y_ion
prec
y2+
y4+
y5++
b4+
y3+
y5+
b2+
b3++
y1+
y4++
TGDKPR+XL_L
y2+
y4+
y3+
b2+
y5++
b4+
y5+
y1+
y4++
KIIQSAK+XL_S
B_b_ion
B_y_ion
y5+
b2+
b4++
y3+
prec
y4+
y1+
y2+
b5+
y6+
KIIQSAK+XL_L
y5+
b2+
prec
y1+
y2+
y3+
b4+
y6+
m/z
